# Compounds of *Citrus medica* and *Zingiber officinale* for COVID-19 inhibition: in silico evidence for cues from Ayurveda

**DOI:** 10.1186/s43094-020-00171-6

**Published:** 2021-01-09

**Authors:** M. Haridas, Vijith Sasidhar, Prajeesh Nath, J. Abhithaj, A. Sabu, P. Rammanohar

**Affiliations:** 1 Inter University Centre for Bioscience and Department of Biotechnology & Microbiology, Dr Janaki Ammal Campus Thalassery, Kannur University, Palayad, 670661 India; 2 Sree Krishna Ayurveda Chikitsa Kendram, Vaikom, Kerala India; 3 Amrita School of Ayurveda, Amrita Vishwa Vidyapeetham, Kollam, Kerala India

**Keywords:** Ayurvedic formulation, COVID-19, SARS-CoV-2 spike protein, Angiotensin-converting enzyme 2, In silico evidence

## Abstract

**Background:**

The nasal carriage of SARS-CoV-2 has been reported as the key factor transmitting COVID-19. Interventions that can reduce viral shedding from the nasopharynx could potentially mitigate the severity of the disease and its contagiousness. Herbal formulation of *Citrus medica* and *Zingiber officinale* is recommended in an Ayurvedic text as a nasal rinse in the management of contagious fevers. These herbs are also indicated in the management of respiratory illnesses and have been attributed with activity against pathogenic organisms in other texts. Molecular docking studies of the phytocompounds of *C. medica* and *Z. officinale* were done to find out whether these compounds could inhibit the receptor binding of SARS-CoV-2 spike protein (S protein) as well as the angiotensin-converting enzyme 2 (ACE-2), as evidenced from their docking into binding/active sites.

**Results:**

The proteins of SARS-CoV-2, essential for its entry into human cells and highly expressed in the goblet and ciliated cells of nasal epithelium, play a significant role in contagiousness of the virus. Docking studies indicated that the specific compounds present in *C. medica* and *Z. officinale* have significant affinity in silico to spike protein of virus and ACE-2 receptor in the host.

**Conclusion:**

In silico studies suggest that the phytochemical compounds in *C. medica* and *Z. officinale* may have good potential in reducing viral load and shedding of SARS-CoV-2 in the nasal passages. Further studies are recommended to test its efficacy in humans for mitigating the transmission of COVID-19.

## Background

The COVID-19 pandemic has become widespread, and the total number of cases in the world has crossed the seven million mark. Transmission of the disease by asymptomatic individuals as well as increased transmission in health care settings is a matter of great concern. A sudden spike in cases, especially severe presentations of COVID-19, overwhelms the health care system. There is a need to explore the potential of multiple interventions in mitigating the transmission and severity of COVID-19. Virus-infected symptomatic patients and upper respiratory tract of SARS-CoV-2 patients with high viral loads are the crucial factors contributing to its high transmission [1]. Several research papers have recommended the use of different solutions for oropharyngeal wash and nasal irrigation as well as a steam inhalation to reduce viral load in the oropharynx and nasopharynx [2, 3]. The Ayurvedic text Yogaratnākara [4] prescribes a herbal formulation with *Citrus medica* and *Zingiber officinale* for nasal cleansing in the context of the treatment of sannipātajvara (fever caused by disturbance of all three doṣas, the human groupings by Ayurvedic constructs) [4].

In COVID-19, high titres of SARS-CoV-2 are detectable in the upper respiratory tract of asymptomatic and symptomatic individuals. The proteins of SARS-CoV-2, essential for its entry into human cells, are highly expressed in the goblet and ciliated cells of the nasal epithelium [1]. Analysis of saliva of COVID-19 patients at the time of admission to the hospital revealed up to 1.2 × 108 infective copies/mL of the virus [5]. It has recently been found that human nasopharynx has a higher viral load than oropharynx [5, 6].

A pilot randomised controlled study of hypertonic saline for nasal irrigation and gargling to treat upper respiratory tract infections in adults has been analysed post hoc. The above treatment was found useful to a subgroup with alpha and beta coronavirus infection, in the reduction of disease symptoms and illness duration [2]. Epithelial cells mount an antiviral effect with hypochlorous acid (HOCl) produced from chloride ions. Epithelial cells have the innate antiviral immune mechanism to clear viral infections. In the presence of chloride ions supplied via salt, enveloped or non-enveloped DNA and RNA viruses were inhibited in epithelial cells [7]. As an adjunct to personal protective equipment, nasal spray and oropharyngeal wash with the povidone-iodine solution in health care workers and patients have been described to reduce the risk of COVID-19 spreading [3]. Thus, the use of nasal cleansing solution has a role to play in controlling the spread of COVID-19 and mitigating severity of the disease.

S protein and ACE-2 are critical in cellular entry and multiplication of SARS-CoV-2 found in COVID-19. Host cell entry of the virus is prerequisite for infection. During this process, S protein recognises host cell receptors and induces viral and host cell membrane fusion.

It is found that the S protein of SARS-CoV-2 is similar to the S protein of SARS-CoV [8]. Substantial structural rearrangement of the S protein from its metastable prefusion conformation to another distinct conformation is required for the fusion of viral envelope with the host cell membrane [9]. If this transformation is curtailed, the virus will become unable to make cell entry and hence cannot cause infection. The irregularly structured connector of the receptor-binding domain (RBD) of S protein (S1) would act as a hinge to engage the cellular membrane by drastic conformational movements. Anything which hinders this process would disable the spike protein to infect the host cell.

Consequently, it may be hypothesised that a viral neutralising agent may have some constituent to bind onto the RBD. The nasal rinse prepared from *C. medica* and *Z. officinale* may likely have ligands with affinity to the RDB capable of making the virus inefficient to infect the host cell. It has been demonstrated that ACE-2 is expressed mainly in alveolar epithelial cells. It is almost absent in other lung cells and also in bronchial epithelial cells, endothelial cells, fibroblasts, and macrophages [10]. Blocking of the renin-angiotensin signal pathway could alleviate severe acute lung injury caused by SARS-CoV-2 S protein, which would otherwise lead to pathogenesis facilitated by ACE-2 receptor [11–13]. An inhibitor of ACE-2 enzyme would, therefore, inhibit the pathogenesis of the COVID-19.

## Methods

### The ligands

**Fig. 1:**
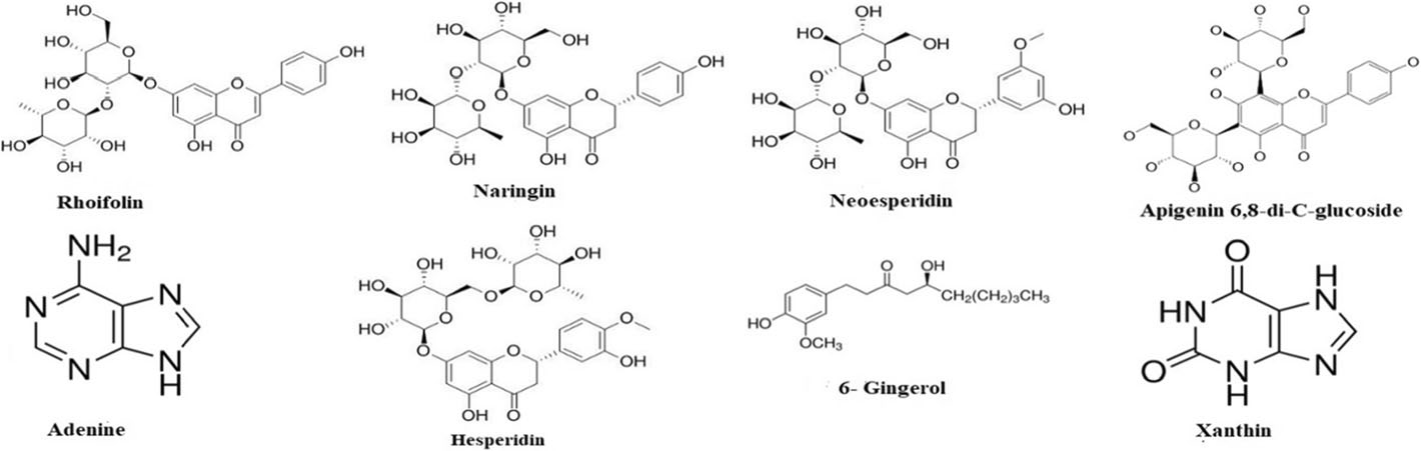
2D structures of COVID-19 spike protein ligands

The herbal constituents of the solution mentioned in the Ayurvedic classic for nasal and throat cleansing for the symptoms similar to COVID-19 are cedrat (*Citrus medica*) and ginger (*Zingiber officinale*) [4, 14]. The compounds rhoifolin, naringin, neohesperidin, apigenin 6,8-di-C-glucoside, adenine, hesperidin, 6-gingerol, xanthin, 8-gingerol, scopoletin, isovanillin, 10-gingerol, 10-shogaol, 8-paradol, and 10-paradol present phenomenally in ginger and cedrat were selected (Figs. 1 and 3) [15, 16].

### The targets

The structure of spike glycoprotein showed as a single polypeptide chain with 1300 amino acids forming a homo-trimer and cleaved by furin-like proteases of the host cell into SI and S2 subunit. S1 subunit is the N-terminal domain which is responsible for the host cell attachment by binding to the cell membrane receptors through its receptor-binding domain, and S2 is the C-terminal domain [17, 18]. The receptor-binding domain in the S1 subunit of the SARS-CoV-2 is therefore responsible for the zoonotic transmission, recognition of host cell, and invasion. The S2 subunit comprises two heptad regions (HR1 and HR2) and a lipophilic fusion peptide and forms the coiled helix structure of the subunit. During the process of infection, the RBD in the S protein bound to the cell surface receptor would trigger conformational change in the S1 and S2. As the consequence of the RBD triggered conformational change, fusion loop exposes and fuses to the membrane together with the heptad region of the S protein, forming a bundle fusion core. These orchestrated molecular movements would facilitate the fusion of viral and host cell membranes. A thorough understanding of the role of ACE-2 and pathophysiological changes caused by the virus in the human body may help discover and explain the corresponding clinical phenomena.

### Protein preparation and receptor grid generation

The downloaded PDB coordinates of SARS-CoV-2 S protein and ACE-2 (6VSB and 1R4L, respectively) were used to prepare the structure by protein preparation wizard in the Maestro software (Maestro, v11.4, Schrodinger, LLC, NY). Since the ligand-bound structure of spike protein was not readily available, the grid preparation was based on the receptor-binding site residues of the spike protein. In the pre-processing step, the hetero group having bond order and formal charges were added, hydrogen atom was added to all the atoms in the system, disulfide bond and zero-order bonds to metals were created, and water molecule within 5 Å in the hetero groups was removed. Then, the structure refinement step followed. The missing side-chain atoms were incorporated. Then for each structure, a brief relaxation was performed with Impact Refinement module (Impref) in order to remove the crystallographic bias. Here, an all-atom constrained minimization was carried out with the aid of forcefield OPLS_2005 to relieve steric clashes present in the original PDB structure. Minimization job will get terminated when the RMSD cutoff of 0.30 Å is attained. Receptor grid was generated based on the binding site residues, where no bound ligand is present. The receptor-binding site residues of SARS-CoV-2 spike protein are TYR449, TYR453, ASN487, GLY496, THR500, GLY502, and TYR505. They were selected for grid generation [19]. ACE-2 grid generation was carried out by selecting the centroid of XX5 (co-crystallised ligand of PDB ID: 1R4L). The receptor grid generation was accomplished by receptor grid generation panel on the GLIDE module of Maestro.

### Ligand preparation

The ligands identified as described above were prepared for using the LigPrep module with Epik to expand protonation and tautomeric states at neutral pH (7 ± 1). The energy minimization of ligands also carried out with OPLS_2005 forcefield.

### Molecular docking

In order to identify the nature of the interaction of the selected compounds with the spike protein, molecular docking was done. GLIDE (Grid-based Ligand Docking with Energetics) module of Schrodinger suite was used for molecular docking. The amino acids of the binding site were mapped. A grid box was generated around the site cavity with a size of 20 Å^3^. The results were expressed based on the binding affinity and ranked by GlideScores (G score), the ligand binding free energy. The initial protocol employed for molecular docking was the standard precision (SP) docking module of GLIDE. Finally, the extra precision (XP) docking method was used to identify the best hits. The G score obtained via ligand-receptor interaction was calculated as implemented in the GLIDE. Top-scoring phytochemicals were subjected for binding free energy prediction by MMGBSA method using Prime Module of the same software.

## Results

Phytocompounds showing good binding affinity towards spike protein are shown in Fig. 1 and for ACE-2 in Fig. 3. The GLIDE scores of the ligand molecules docked on to the SARS-CoV-2 spike protein are listed in Table 1. The binding energy of these compounds docked onto the protein is calculated by the method Prime Module of the same software which has been given as Table 2.

**Table 1:** Molecular docking results of the COVID-19 spike protein and the ligands from the Ayurvedic preparation [4]

Compound	Plant	GlideScore
Rhoifolin	*C. medica*	− 7.361
Naringin	*C. medica*	− 7.112
Neohesperidin	*C. medica*	− 6.564
Apigenin 6,8-di-C-glucoside	*C. medica*	− 6.409
Adenine	*Z. officinale*	− 5.004
Hesperidin	*C. medica*	− 4.791
6-Gingerol	*Z. officinale*	− 4.177
Xanthin	*C. medica*	− 4.063

**Table 2:** Spike protein binding free energies of top-scoring phytocompounds

Compound name	H bonds	Interacting residues	Binding free energy
Rhoifolin	5	TYR453, SER494, TYR495, THR500	− 58.43
Naringin	5	TYR453, SER494, TYR499, THR500	− 44.48
Neoesperidin	5	TYR453, SER494, GLY494, GLY504	− 60.95
Apigenin 6,8-di-C-glucoside	5	TYR449, TYR453, GLY496, GLY504	− 37.87
Adenine	2	TYR495, GLY496	− 24.18
Hesperidin	2	GLY496, TYR505	− 59.47
6-Gingerol	4	TYR453, SER494, GLY496, TYR505	− 38.60
Xanthin	1	TYR453	− 18.26

The GLIDE scores show the affinity of the docked compounds to the binding site (ACE-2 receptor) of the SARS-CoV-2 spike glycoprotein. Table 1 lists eight compounds in the decreasing order of the binding affinity towards the spike protein. The lower-ranking compounds are omitted for convenience. The ranking was done on the basis of GLIDE score, taking − 5.00 kcal/mol as an appropriate benchmarking critical score for the top-scoring compounds namely rhoifolin, naringin, neohesperidin, and apigenin 6,8-di-C-glucoside. They showed critical interaction with TYR453 which is an important residue present in the RBD domain of spike protein. Without reference to an experimental value of a standard inhibitor, it will be inappropriate to make a quantitative statement on the behaviour of the SARS-CoV-2 spike glycoprotein binding molecule. However, it could be understood that there are many compounds which would bind to the SARS-CoV-2 spike glycoprotein and would render it incapable of interacting with the host cell membrane to initiate pathogenesis. Table 2 also shows an explanatory binding energy profile of the same compounds towards the spike protein.

Apart from the protein binding free energies of top-scoring phytocompounds, this table shows their contacts to the protein. There are differences in the contact residues for different compounds. It is understood as their chemical differences in interacting moieties.

**Fig. 2:**
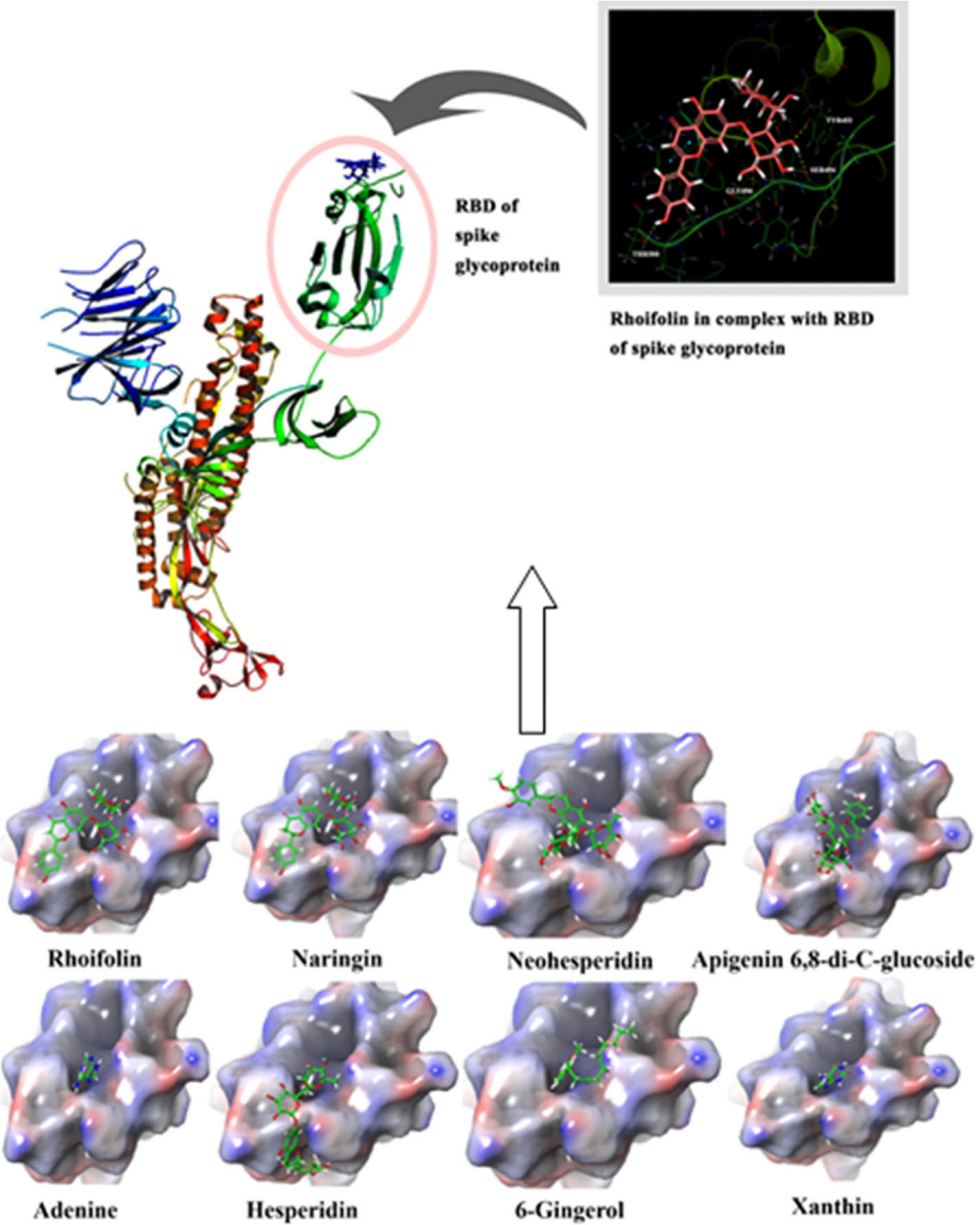
Docked postures of the phytocompounds onto the receptor-binding site of the SARS-CoV-2 spike glycoprotein

Figure 2 shows the protein-ligand interactions of all the eight compounds taken up for study as surface views. Also, the protein binding details of the top-scoring compound have been shown as a molecular cartoon. Protein-ligand interaction, at the receptor-binding site of the protein, of rhoifolin having the highest GLIDE score is shown in detail in the figure. Table 2 gives the details of interacting residues of the target protein with rhoifolin.

**Fig. 3:**
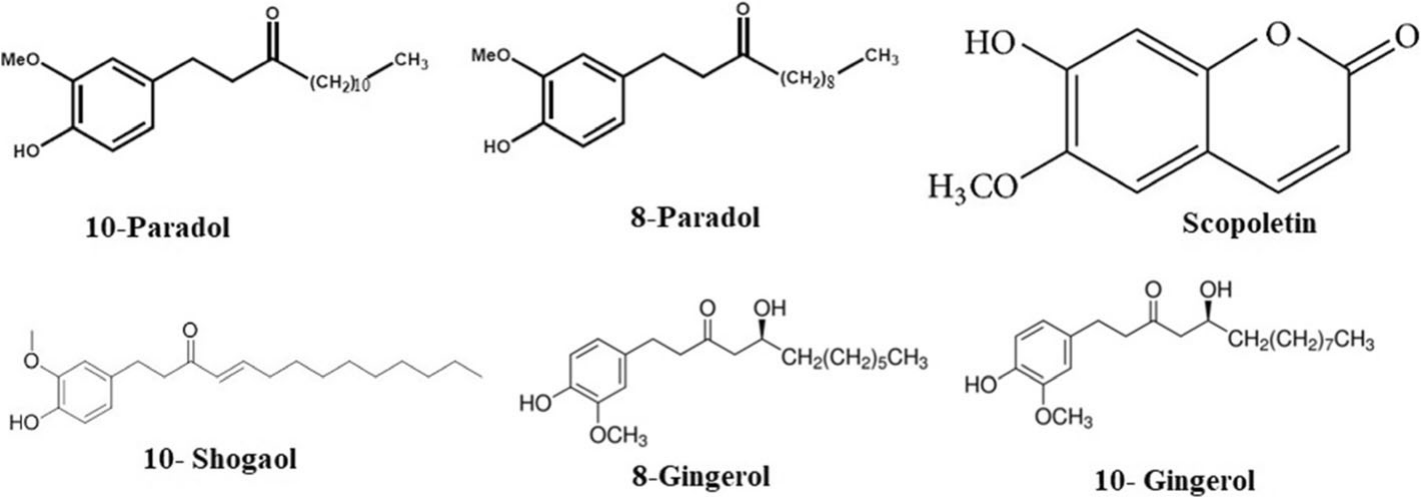
2D structures of ACE-2 ligands (2D structures of hesperidin and neohesperidin are shown in Fig. 1)

**Fig. 4:**
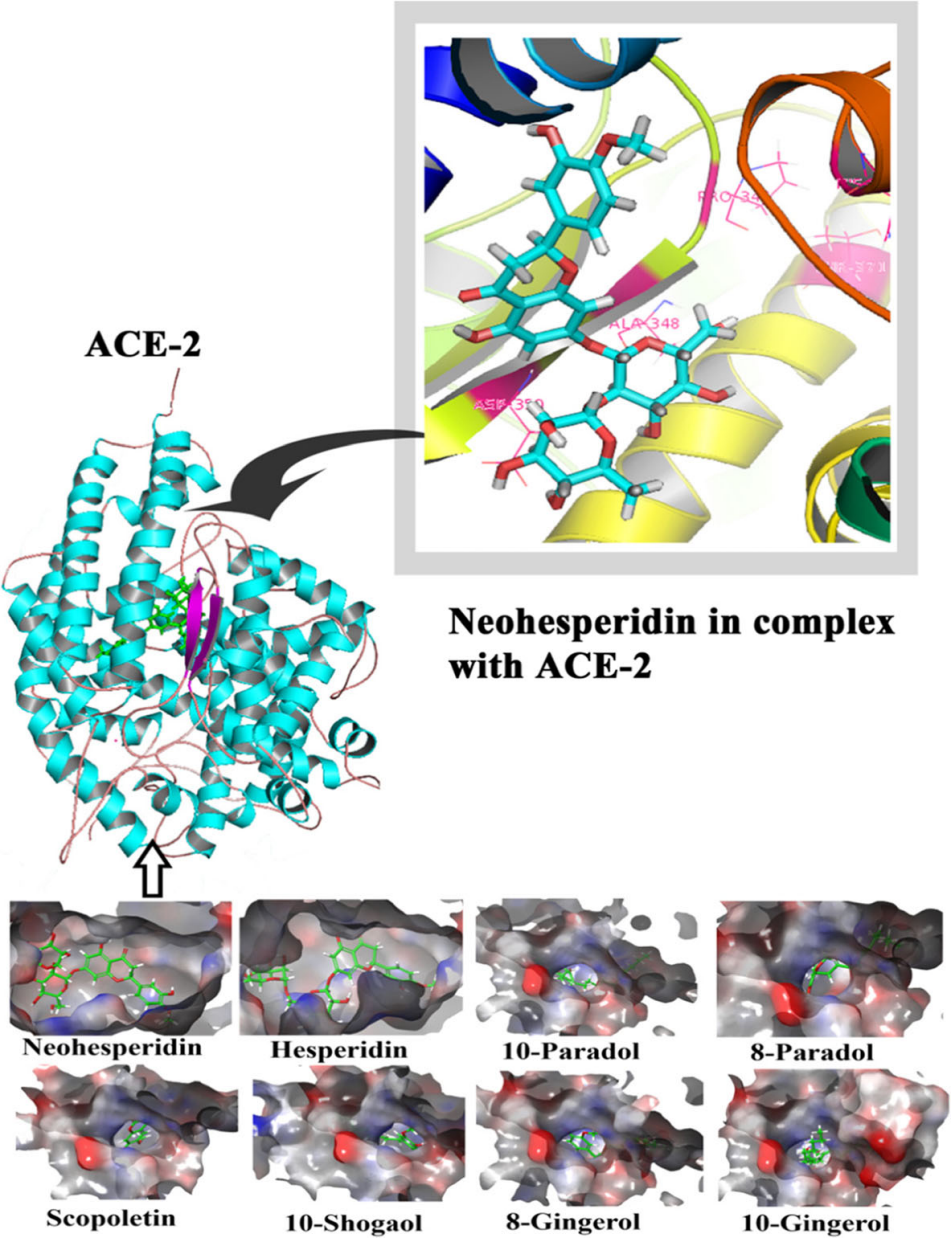
Docked postures of the phytocompounds onto the active site of the ACE-2

The GLIDE scores of the ligand molecules docked on to the ACE-2 are listed in Table 3. The binding energy of these compounds docked onto the protein is calculated by the method Prime Module of the same software which has been given as Table 4. Like in the previous case of the spike protein-ligand interactions, the GLIDE scores in the case of ACE-2 at its RBD show the affinity of the docked compounds. Table 3 lists eight compounds in the decreasing order of the binding affinity towards the ACE-2 receptor. The compound found co-crystallised with ACE-2 was also considered for docking exercise. The lower-ranking compounds are omitted for convenience since they would tend to insignificance. It may be seen that the compound, docked into the active site of ACE-2 during co-crystallisation, ranks only as of the lowest amongst the listed compounds. It shows that all the other eight compounds may be more potent inhibitors to the ACE-2. It could be discerned that there are many compounds which would bind to the ACE-2 and would render it inactive for the pathogenesis of COVID-19 (Fig. 3). Figure 4 shows the protein-ligand interactions of all the eight compounds taken up for study as surface views. Details of the protein binding with the top-scoring compound have been shown as a molecular cartoon. Protein-ligand interaction, at the active site binding of the enzyme, of neohesperidin having the highest GLIDE score is shown in detail in the figure. Neohesperidin forms three H bonds with ASN51, ALA348, and ASP350 present on the ACE-2 and has the binding energy of − 51.48 kcal/mol.

**Table 3:** Molecular docking results of the ACE-2 and the ligands from the Ayurvedic preparation [4] and the co-crystallised compound

Compound	Plant	GlideScore
XX5*	Standard	− 4.36
Neohesperidin	*C. medica*	− 9.94
Hesperidin	*C. medica*	− 8.28
10-Paradol	*Z. officinale*	− 6.11
8-Paradol	*Z. officinale*	− 4.76
Scopoletin	*Z. officinale*	− 4.73
10-Shogaol	*Z. officinale*	− 4.70
8-Gingerol	*Z. officinale*	− 4.69
10-Gingerol	*Z. officinale*	− 4.56

XX5*—(S,S)-2-{1-Carboxy-2-[3-(3,5-Dichloro-Benzyl)-3h-Imidazol-4-Yl]-Ethylamino}-4-Methyl-Pentanoic Acid

**Table 4:** Binding free energies of top scored compounds and control at the active site of ACE-2

Compound name	No. of H bonds	Interacting residues	Binding free energy
XX5*	3	ASP367, LYS363, ASN368	− 31.39
Neohesperidin	3	ASN51, ALA348, ASP350	− 51.48
Hesperidin	4	TRP203, ASN394, SER511	− 59.87
10-Paradol	2	ARG273, LYS363	− 37.87
8-Paradol	3	ASN149, LYS363	− 51.27
Scopoletin	3	ASN149, ASN368, LYS363	− 40.32
10-Shogaol	3	ASN277, LYS363, ASP367	− 44.61
8-Gingerol	3	ASP367, LYS363	− 46.85
10-Gingerol	2	GLU406, ARG518	− 33.72

## Discussion

The in silico evidence from our study suggests that the Ayurvedic nasal purge [4] could inactivate the virally transcripted S protein required for pathogenesis. Besides, it also inhibits the ACE-2 receptor in the host cell. The docking studies implicate that the mode of action of the Ayurvedic nasal purge simulates the use of a subunit vaccine by the mechanism of action lasting for a short time.

The Ayurvedic text clarifies that by the use of this nasal purge, the mucus secretions will flow out relieving the afflictions of the head, chest, throat, mouth, and the sides of the chest [4]. It indicates that the purpose of this application is to protect the organs connected to the upper respiratory passages and mitigate the severity of the symptoms. Nasya is also recommended as a preventive practice as part of the daily routine for nasal hygiene. Suśrutasamhitā also advises the application of nasal drops by a healthy person before going out of one’s house into the public spaces [20].

In the formulation used in our study, fresh ginger and cedrat are the primary ingredients along with rock salt, black salt, and ammonium chloride. The ionic salts may help the absorption of the compounds present in the medicinal preparation, essentially derived from the two major plant materials mentioned above. This formulation is used for cleansing the nasal passages to obtain relief from symptoms of sannipātajvara. Certain other citrus fruits also could be used to treat cough and dyspnoea, and cleanse the throat, tongue, and heart [21]. Its ability to clear the nasopharynx and oropharynx has also been emphasised in anther classic text [22]. Also, it is attributed to antimicrobial and anti-inflammatory activity [23]. Ginger has been attributed to the anti-inflammatory activity. It is also indicated for the management of respiratory disorders like dyspnoea and cough [24]. The phytocompounds in this formulation will likely have benefit in reducing the severity of COVID-19 by inhibiting SARS-CoV-2 in the nasal passages. Such a formulation would be relevant for application in individuals with high-risk exposure as well as COVID-19 patients who are asymptomatic or have mild symptoms to mitigate the transmission of SARS-CoV-2. We examined in silico target binding behaviour of the major phytochemical components of both ginger and cedrat to find out if they could inhibit the critical treatment targets of COVID-19, the spike protein and the ACE-2 receptor. Besides the above hypothesis, the hypertonic saline solution and fresh ginger juice also have antiviral activity [24]. Effects of fresh and dried ginger hot water extracts on HRSV, in human upper (HEp-2) and lower (A549) respiratory tract cell lines, were tested by plaque reduction assay [25]. The ability of ginger to stimulate antiviral cytokines was evaluated by ELISA [14]. Fresh, but not dried, ginger is effective against HRSV-induced plaque formation on airway epithelium by blocking viral attachment and internalisation [25]. However, the above studies reporting positive results of the antiviral effect of ginger have not explained the mechanism of action at the level of molecular interactions. As hypothesised, the compounds present in ginger and cedrat may have neutralising effect on SARS-CoV-2 by inhibiting the spike glycoprotein in the virus and the enzyme ACE-2 in the host, both being crucial factors enabling cell entry of SARS-CoV-2. As a preliminary step to test this hypothesis, the interactions at the active/binding site of the SARS-CoV-2 with the phytocompounds present in ginger and cedrat by molecular docking were studied.

Chang et al. [25] have shown that only fresh ginger inhibited human respiratory syncytial virus-induced plaque formation in HEp-2 and A549 cell lines dose-dependently. They have shown that fresh ginger inhibited viral attachment and internalisation dose-dependently. Fresh ginger of high concentration could stimulate mucosal cells to secrete interferon-β that possibly contributed to counteracting viral infection. Fresh ginger stimulates secretions more effectively compared to dry ginger [14]. Also, Greño et al. [26] have demonstrated that *Citrus medica* also has some antiviral properties. However, these are not explained in molecular terms. The molecular mechanism of antiviral property of certain compounds found in ginger and cedrat has been demonstrated here in silico. It may also be noted that there may be other biological mechanisms underlying the antiviral action of the medicinal preparations containing ginger and cedrat. It is not clear whether the compounds in the Ayurvedic formulation [4] discussed above will enter the systemic circulation or other areas in the upper and lower respiratory tracts. It is quite possible that the well-known anti-inflammatory activity of ginger may also have a role in giving relief from symptoms of ‘sannipatajvara’ in the head, oral cavity, throat, and chest as claimed in the Yogaratnakara [4]. Further studies exploring such activities of the formulation assume relevance in the present context of COVID-19. As ginger in higher doses can irritate the mucous membranes, the dosage and dilution of the formulation must be optimised. Especially, the combination of ginger has been contraindicated in skin diseases, in bleeding disorders, and in summer. These contraindications also apply to salt [22].

There are limitations to this study, and the in silica evidence needs to be substantiated by possible in vitro and human clinical studies. Before human studies, the formulation has to be standardised. As the formulation contains ingredients like ginger, the possibility of insults to the delicate nasal epithelium must be kept in mind while optimising the dosage.

## Conclusion

In the absence of a vaccine, the contagiousness of SARS-CoV-2 virus is a matter of primary concern. COVID-19 pandemic continues to spread across the length and breadth of the world. The use of an ancient, time-tested formulation for nasal cleansing could play an important role in mitigating the contagiousness of SARS-CoV-2 by inhibiting the multiplication and shedding of the virus in the nasal epithelium. Administration of this nasal purge may be especially relevant in people with high-risk exposure to COVID-19 patients like frontline workers and health care professionals. It could also be helpful in asymptomatic patients and patients with mild symptoms in the early stages of the disease, when viral shedding and contagion are highest. It may be summed up that the administration of nasal purge could be considered for in vitro studies and human trials for mitigating transmission and severity of COVID-19. As demonstrated in silico, most of the major chemicals found in cedrat and ginger do interact at the active sites of the RBD of COVID-19 spike protein and human ACE-2, to elicit antiviral property and inhibit spreading of COVID-19 disease. This would become an ideal home remedy for COVID-19 disease in the present context.

## Data Availability

All data generated or analysed during this study are included in this published article [and its supplementary information files].

## Abbreviations

SARS-CoV-2: Severe acute respiratory syndrome coronavirus 2
COVID-19: Coronavirus disease 2019
ACE-2: Angiotensin-converting enzyme 2
HOCl: Hypochlorous acid
RBD: Receptor-binding domain
S protein: Spike protein
HR1 and HR2: Heptad regions 1 and 2
PDB: Protein Data Bank
RMSD: Root mean square deviation
GLIDE: Grid-based Ligand Docking with Energetics
G score: GlideScores
SP: Standard precision
XP: Extra precision

## References

[1] Sungnak W, Huang N, Bécavin C, Berg M, Queen R, Litvinukova M, Talavera-López C, Maatz H, Reichart D, Sampaziotis F, Worlock KB, Yoshida M, Barnes JL (2020). SARS-CoV-2 entry factors are highly expressed in nasal epithelial cells together with innate immune genes. Nat Med.

[2] Ramalingam S, Graham C, Dove J, Morrice L, Sheikh A (2020). Hypertonic saline nasal irrigation and gargling should be considered as a treatment option for COVID-19. J Glob Health.

[3] Kirk-Bayley J, Challacombe S, Stephen Sunkaraneni VS, Combes J (2020). The use of povidone iodine nasal spray and mouthwash during the current COVID-19 pandemic may reduce cross infection and protect healthcare workers.

[4] Shastri S (2005). Yogaratnakara.

[5] Zou L, Ruan F, Huang M, Liang L, Huang H, Hong Z, Yu J, Kang M, Song Y, Xia J, Guo Q, Song T, He J, Yen HL, Peiris M, Wu J (2020). SARS-CoV-2 viral load in upper respiratory specimens of infected patients. N Engl J Med.

[6] Zhou P, Yang XL, Wang XG, Hu B, Zhang L, Zhang W, Si HR, Zhu Y, Li B, Huang CL, Chen HD, Chen J, Luo Y, Guo H, Jiang RD, Liu MQ, Chen Y, Shen XR, Wang X, Zheng XS, Zhao K, Chen QJ, Deng F, Liu LL, Yan B, Zhan FX, Wang YY, Xiao GF, Shi ZL (2020). A pneumonia outbreak associated with a new coronavirus of probable bat origin. Nature.

[7] Ramalingam S, Cai B, Wong J, Twomey M, Chen R, Fu RM, Boote T, McCaughan H, Griffiths SJ, Haas JG (2018). Antiviral innate immune response in non-myeloid cells is augmented by chloride ions via an increase in intracellular hypochlorous acid levels. Sci Rep.

[8] Xu X, Chen P, Wang J, Feng J, Zhou H, Li X, Zhong W, Hao P (2020). Evolution of the novel coronavirus from the ongoing Wuhan outbreak and modeling of its spike protein for risk of human transmission. Sci China Life Sci.

[9] Wrapp D, Wang N, Corbett KS, Goldsmith JA, Hsieh CL, Abiona O, Graham BS, McLellan JS (2020). Cryo-EM structure of the 2019-nCoV spike in the prefusion conformation. Science.

[10] Zou X, Chen K, Zou J, Han P, Hao J, Han Z (2020) Single-cell RNA-seq data analysis on the receptor ACE2 expression reveals the potential risk of different human organs vulnerable to 2019-nCoV infection. Front Med. 10.1007/s11684-020-0754-0 10.1007/s11684-020-0754-0 PMC7088738 32170560

[11] Kuba K, Imai Y, Rao S, Gao H, Guo F, Guan B, Huan Y, Yang P, Zhang Y, Deng W, Bao L, Zhang B, Liu G, Wang Z, Chappell M, Liu Y, Zheng D, Leibbrandt A, Wada T, Slutsky A, Liu D, Qin C, Jiang C, Penninger JM (2020). A crucial role of angiotensin converting enzyme 2 (ACE2) in SARS coronavirus-induced lung injury. Nat Med.

[12] Imai Y, Kuba K, Rao S, Huan Y, Guo F, Guan B, Yang P, Sarao R, Wada T, Leong-Poi H, Crackower MA, Fukamizu A, Hui CC, Hein L, Uhlig S, Slutsky AS, Jiang C, Penninger JM (2005). Angiotensin-converting enzyme 2 protects from severe acute lung failure. Nature.

[13] Li S, Tang Z-j, Li Z, Liu X (2020) Searching therapeutic strategy of new coronavirus pneumonia from angiotensin-converting enzyme 2: the target of COVID-19 and SARS-CoV. Eur J Clin Microbiol Infect Dis. 10.1007/s10096-020-03883-y 10.1007/s10096-020-03883-y PMC7152693 32285293

[14] Harisadasiva S (2011). Astangahridayam.

[15] Chhikara N, Kour R, Jaglan S, Gupta P, Gat Y, Panghal A (2018) *Citrus medica*: nutritional, phytochemical composition and health benefits-a review. Food Funct. 10.1039/C7FO02035J 10.1039/c7fo02035j 29594287

[16] Mukkavilli R, Yang C, Singh Tanwar R, Ghareeb A, Luthra L, Aneja R (2017). Absorption, metabolic stability, and pharmacokinetics of ginger phytochemicals. Molecules.

[17] Lomniczi BJ (1977). Biological properties of avian coronavirus RNA. J Gen Virol.

[18] Lee HJ, Shieh CK, Gorbalenya AE, Koonin EV, LaMonica N, Tuler J, Bagdzhadzhyan A, Lai MM (1991). The complete sequence (22 kilobases) of murine coronavirus gene 1 encoding the putative proteases and RNA polymerase. Virology.

[19] Lan J, Ge J, Yu J, Shan S, Zhou H, Fan S, Zhang Q, Shi X, Wang Q, Zhang L, Wang X (2020). Structure of the SARS-CoV-2 spike receptor-binding domain bound to the ACE2 receptor. Nature.

[20] Acarya YT (2008). Susrutasamhita of Susruta.

[21] Shastry VD (1994). Bhavaprakasha Nighantu.

[22] Dash B (2001). Madanapala Nighantu.

[23] Singh A (2008). Dhanvantari Nighantu.

[24] Sharma PV, Guruprasad (1979). Kaiyadeva Nighantu.

[25] Chang J, Wang K, Yeh C, EnShieh D, Chiang L (2013). Fresh ginger (*Zingiber officinale*) has anti-viral activity against human respiratory syncytial virus in human respiratory tract cell lines. J Ethnopharmacol.

[26] Greño V, Cambra M, Navarro L, Durán-Vila N (1990). Effect of antiviral chemicals on the development and virus content of citrus buds cultured in vitro. Sci Hortic.

